# Supplementary material for: Are carbon emissions trading and green financial instruments synergistic? -Comprehensive quantitative research based on content analysis
Source: PLoS One. 2024 Mar 7;19(3):e0298601. doi: 10.1371/journal.pone.0298601 (PMC10919601; doi:10.1371/journal.pone.0298601)
Supplement: S1 Data — (ZIP) [file pone.0298601.s001.zip › Data/地级市Z_F工作报告.zip!/║┌┴·╜¡/╦τ╗»╩╨/└┤╫╘╣1⁄2╓┌║┼í╛┬φ┐╦╩2╛▌═°í┐_2009.docx]

各位代表：

现在，我代表市政府向大会作政府工作报告，请审议，并请列席会议的同志提出意见。

一、2008年工作回顾

过去的一年，我们在省委、省政府和市委的领导下，以科学发展观为统领，秉持发展为上、民生为本、务实为先、落实为重的执政理念，解放思想，创新发展，团结一心，攻坚克难，各项工作都取得了较好成绩，圆满完成了三次会议确定的各项任务。

——经济增长成果喜人。省考核的9项经济指标全部达标，其中6项指标增速超上年、超预期、超全省平均水平，实现了历史性增长、突破性增长。地区生产总值534.2亿元，同比增长14.9%，高于全省3.1个百分点；城镇固定资产投资138.7亿元，增长48.7%，高于全省13.5个百分点，为历年最高增幅；全口径财政收入34.2亿元，增长26.9%，一般预算收入15.9亿元，增长32.1%，高于全省0.8个百分点，是财政形势最好的一年；城镇居民人均可支配收入7109元，增长16.9%，高于全省3.9个百分点，农民人均纯收入3765.4元，增长18.3%，高于全省1.3个百分点。农业经济登上新台阶。粮食总产再创新高，达到216.5亿斤，纯增45.8亿斤。肇东荣获全国产粮大县标兵，海伦、望奎、青冈、北林被评为全国产粮大县；畜牧业增加值实现73亿元，占农业增加值比重上升到41%；完成造林22万亩，继续保持全省领先。工业经济提速增效。规模以上工业增加值、主营业务收入、利税分别增长19.3%、24.5%和32.2%，均高于全省平均增幅；食品、医药、化工等主导产业不断壮大，支撑作用进一步增强；万元规模以上工业增加值和地区生产总值能耗分别下降6%和3.6%。商贸经济更趋活跃。社会消费品零售总额186亿元，增长21%；进出口贸易总额7861万美元，增长75.8%，高于全省43.4个百分点，增幅位居全省第二；实际利用外资3618.4万美元，增长25.3%，连续6年创历史新高。非公经济快速发展。所占经济总量比重上升到47.5%，民营企业发展到6700 多户。10个县（市）区全面大上，肇东跻身全国百强，安达稳居全省十强，4个中等县份晋档升级，4个“十弱县”超常发展，提速增效中加快了跨越追赶的步伐。

——招商引资规模空前。项目开发建设硕果累累。引进具有一定规模的项目286个，合同引资131亿元，到位66.1亿元，同比增长33%和41.7%。新建续建投资超3000万元项目170个，完成投资86.4亿元，纯增38.4亿元，增幅高达80%。大项目数量成倍增长，引进新安工业硅、申禄达国际家居建材城、杰隆生物、天津宝迪、杭州贝因美二期、南华糖业扩产、阳达大豆、双汇北大荒、朝阳亚麻染整、嵩天马铃薯蛋白、龙凤热电联产、格林谷氨酸等超亿元项目49个，是上年的3.3倍。项目结构质量更趋优化，工业项目占到60%以上，稻壳发电、米糠制油、大豆浓缩蛋白、动物血蛋白、延迟焦化等一批科技含量高、循环效益好、产业配套性强的精深加工项目取得实质性突破；以家居建材、物流配送、商业城、旅游等为代表的大市场、大商贸和现代服务项目快速兴起，正在改变着传统的商贸结构。项目集聚性增强，80%以上的项目落户到工业集聚区，全市17个工业集聚区累计投入基础建设资金7亿元。招商引资火热寒地黑土，以项目扩总量、调结构、增财税，集聚了后劲，增强了活力，更加坚定我们的发展信心。

——基础建设全面推进。2008年，我市城乡基础建设投入力度大、面貌变化大。市县两级城市基础建设完成投资13.75亿元，比上年增加近1倍。大力实施了硬化、绿化、亮化、美化、给排水、商业街改造、污水处理、垃圾处理和集中供热等重点工程。新建高级、次高级道路131公里，新修巷路150公里；新增集中供热面积482万平方米，集中供热覆盖率达到48%，高出全省3个百分点；市本级、肇东、安达3个城市污水处理项目顺利开工建设，投资3.2亿元，新上改建工业废水处理设施45处。农村基础建设完成投资37.8亿元，是历史上投入最高的一年。修建农村公路5366公里，是计划的3.6倍，建设里程连续三年居全省第一；新建饮水安全工程139处，解决了近10万人饮水安全问题；建设户用沼气池、秸秆气化等新型能源工程 1万多处；新建改造农贸市场43个、休闲广场 69 处，农村公共设施明显加强。农业基础建设完成投资15亿元。启动实施了 “庆北绥”200万亩水田现代园区示范项目，建成后每年可以增产粮食6亿斤；新建水稻育秧大棚2.2万座；改造大中型灌区7处，消险加固中小型水库6座，新打机电井6420眼，有效灌溉面积达720万亩，阁山水库和永安灌区渠首二期配套工程顺利立项；新增大型农机具 2677台（套），农机专业合作社发展到 234 个。基础建设的全面加强，必将有力推进我们的全面发展。

——市本级发展步伐加快。财政增长成番论倍。财政总收入11.93亿元，比上年增长76.2%。其中，地方财政收入7.53亿元，增长127%，基金预算收入5.02亿元，增长236%。发展后劲增强。新上续建投资超3000万元项目19个，其中超亿元项目8个，仅投资14.5亿元的工业硅项目，全部达产后就可实现销售收入25亿元、税金2亿元。市经济开发区实现全口径财政收入6000万元，其中企业税收4000万元，均是上年的2倍。市政基础建设全面升级。全年实施市政工程项目230多项，完成投资5.5亿多元，其中财政投资3.5亿元，涉及主巷路改造、给排水配套、园林绿化、街路亮化、公园广场、污水处理、垃圾处理、集中供热等十几个重点工程。“百路”工程全面完成，改造城区道路122条、57.8公里，道路数量、路长、总面积、投资额创下新纪录，将有5万多居民出门不再走泥泞路。实施给排水工程83项，使一些偏僻巷路都有了下水，方便了百姓生活。新增绿化面积33万平方米，新建改造人民广场、拥军广场和人民公园等广场公园10处，市民散步健身有了更多去处。安装路灯987基，新增供热面积180万平方米，供热规模达到520万平方米，又使一大批居民住上了暖屋子。日处理5万吨的污水处理项目顺利开工建设。棚户区改造取得历史性突破。成建制组团式开发改造第一批棚户区项目5个、建筑面积53万平方米，回迁安置面积16万平方米，配建廉租房2.8万平方米，有2596户棚户区居民没花一分钱就实现了破旧平房换新楼房，从而环境好了，路好了，心情更好了。回迁户喜迁新居时的笑容和表情说明他们对棚户区改造的拥护和支持。有700多户低收入家庭住上廉租房，相当于财政拿出补贴资金1.3亿元。启动了第二批棚户区改造项目16个，已有9个区块31万平方米开始拆迁。市区房地产开发进入高峰，开工建设112万平方米，是2007年的3倍，给财政创收1亿多元。碧桂园、秀水嘉园、电力名苑、福乾花园、颐和苑等在建开发项目都在10万平方米以上。大规模的开发建设，用别人的钱改造了我们的城市，拉动了相关十几个产业发展，扩大了就业，增加了税收，使百姓买房、住房有了更多的价格和区位选择。这不仅符合中央和省大发展、快发展的要求，更是本届政府前三年用城市开发拉动增长，后两年用招商上项目拉动增长的宏观战略。投资1.2亿元建成的现代医疗中心，服务功能和水准全省一流。市科技文化馆如期交付使用，使百姓又多了一处科普、娱乐、学习、锻炼的好场所。所有这些发展变化，表明我们市本级的综合实力快速提升，中心城市的功能和形象逐渐显现。

——改善民生成效显著。三次会议提出的关乎民生的10项重点工作全部完成和超额完成。在就业再就业方面，新增就业6.11万人，发放小额担保贷款2546万元，开发公益性岗位3860个，比预期目标分别增6100人、1000万元和860个；内转外输劳动力104万人，创劳务收入58亿元。在社会保障方面，适时提高低保和五保标准，城乡40万低保对象实现应保尽保，对42万低收入群体实施了生活救助，城乡社会救助资金总量比上年增加1倍以上；8750名符合条件的集体企业退休人员实现了领取低保向领取养老金的根本性转变，保障标准提高4倍以上。投资近1亿元，新建县级中心敬老院12处，入住3360人，占集中供养总数的36%，供养标准明显提高。在改善群众医疗服务方面，率先打破政府包办模式，成功与人寿保险公司合作，实施了城市低保对象和低收入群体医疗救助保险，低保对象参保面突破80%。社区卫生服务机构发展到72个，覆盖人口120万人。新建改造乡镇卫生院53处，新农合参合率达到93.3%，超出了省定目标。人口计生工作服务水平进一步提高。在实施安居工程方面，城乡弱势群体住房条件显著改善，“一棚一草”改造取得重大突破。完成棚户区拆迁面积75万平方米，改造建筑面积145万平方米，配建廉租房5万平方米，群众间接受益达5亿多元；投资29.42亿元，改造泥草房5.26万户，改造农村倒危房2130户，均占全省的四分之一。在商品房开发方面，县城以上房地产开发面积311万平方米，人均住房建筑面积达23.85平方米，比上年增加1.5平方米。在扶贫解困方面，投入各类救灾款项2455.6万元，解决了22万灾民和贫困户的基本生活困难。全市人民心系汶川灾区，共捐款3313.8万元。在全省率先完成企业职工解欠工作，解决历史拖欠工资11.5亿元。城乡16万人走出贫困，超出了预期目标。在科教和谐发展方面，组织实施科技计划项目78项；新建改造城乡中小学校27万平方米；投入9120万元，完成2000多所农村中小学现代远程教育工程；落实2.45亿元资金，实现城乡全部免费义务教育。在文化体育方面，新建县以上大型文化场馆4个，新建乡镇文化站15个、村级文化活动室207个；新建县级体育场馆8处，各类全民健身工程142处；全市数字电视全面开通。在社区建设方面，建成1000平方米以上的县级社区服务中心4处、社区服务站161个，社区办公用房自有率达到60%。在平安绥化建设方面，9个县（市）均被评为省级平安县；全年没有发生重特大安全生产事故，连续九年被省政府评为安全生产优秀单位；解决历史信访积案438起；社会治安综合治理不断加强，连续多年获全省优胜荣誉。我们通过改善民生，进一步巩固扩大了和谐社会的创建成果。

此外，民族宗教、审计监察、市场监管、妇女儿童、外事侨务、民兵预备役、双拥优抚、国防人防、残联、气象、档案、地方志等各项工作都取得明显成绩。

回顾一年来的工作，主要体会，一是依靠市委抓发展。时刻把市委的决策、部署作为工作的中心，坚持党的领导，做到方向明、路数清。二是依靠人大、政协及各民主党派、工商联和无党派人士的支持监督抓发展。主动接受监督，充分吸纳各方意见及建议。人大政协高度关注、鼎力支持政府工作，民主党派、工商联、无党派人士及社会各界积极参与、全力支持政府工作。每一项成绩的取得，都是各方积极监督、协调配合、努力支持的结果。三是解放思想抓发展。我们靠解放思想，舍小利、让近利，求大利、争远利，实现了招商引资的新突破；靠解放思想，经营开发城市，用财政投入撬动社会资本，用开明政策吸引各方参与，实现了城市开发轰轰烈烈，基础建设全面大上；靠解放思想，破解难题，化解矛盾，在教育改革、城管体制、医疗救助、社区建设、全民创业等方面积极探索、大胆尝试，着力排除体制机制上的障碍。四是开拓创新抓发展。我们绥化不是煤城，而龙煤集团却能落户绥化，在绥化发展壮大；我们绥化没有硅矿，而硅加工项目却能争先选择绥化，在绥化迅速开工建设；我们绥化欠发达，而在全球经济下行的背景下，我们的经济建设和财政收入却能大幅增长，甚至成番论倍地增长。所有这些，靠的就是创新思维，创新举措，创新发展。五是以民为本抓发展。我们始终把关注民生、改善民生、服务民生作为政府工作出发点和落脚点。积极创造条件，大力发展民生事业，千方百计解决群众关心的行路难、住房难、就医难、就业难等问题。在财力比较紧张的情况下，舍得民生事业投入，全年争取国家和省民生方面的投入至少在20亿元以上，地方财政投入也至少在10亿元以上。六是求真务实抓发展。坚持少争论、多实践，少务虚、多实干，对认定的事紧抓不放，对要干的事一干到底，对难干的事不遗余力。从碧桂园引进到硅工业园建设，从“百路”工程到棚户区改造，从全国百强县到全国产粮大县，从“十弱”县到经济发展相对强县，都是我们真抓实干干出来的，攻坚克难攻出来的。七是优化服务抓发展。就是向服务要效率、向服务要质量、向服务要成果。在市直机关积极组织开展“十清”，成立“马上办”办公室，大力推进政务公开，认真清理各种规费，下决心解决靠权力寻租和以费养人的问题，全市先后停止和暂缓行政审批及收费近二百项，着力改善发展环境。八是完善制度抓发展。政府出资的所有工程项目全部阳光操作，通过完善工程项目预算、严格财政评审、改投标为竞标，既节约了资金，又从机制和制度上避免了以工程、项目、土地谋私的问题。

一年来，从城市到农村，到处都发生着显著变化，有目共睹，令人欣喜。这些成绩的取得凝聚着四大班子的共同心血，汇集着全市人民的力量。在此，我代表市政府，向过去一年里为各项事业发展做出贡献的人们表示感谢！向支持政府工作、关心政府工作的人们表示感谢！

过去的一年，全市经济社会虽然取得了可喜成果，但按照科学发展的要求，用广大人民群众的期盼来衡量，还存在着一些亟待解决的矛盾和问题。一是个别招商引资计划项目没有落实。虽然已在数量上、质量上成倍的完成了招商任务，一年顶多少年，但中粮集团拟投资项目由于国家政策调整，中央领导对中粮集团投资玉米加工项目有明确指示，致使投资不能到位。但中粮集团表示，一旦政策允许，绥化项目就可启动。二是城市管理还有待加强。虽然近年由于修路带来的变化，城市脏乱差问题已有明显改观，但个别区段保持不经常，时好时坏，有死角死面，与群众的期望值比还有差距。三是对政府序列部门需要强化管理。由于工作中注重抓发展，忽略抓管理，使得个别执法单位纪律涣散，管理松弛的旧病不能及时得到整治，产生了一定的不良影响。四是服务环境还有待于加强。一些单位工作作风差、服务水平低，群众还对个别部门的工作不十分满意。五是经济总量小、财政保障能力弱。这仍然是制约经济社会发展的主要问题。对这些问题要高度重视，切实加以解决。

二、2009年的主要任务

各位代表！2009年，风险和机遇交织，压力与挑战并存，形势严峻，任务艰巨。

全球性的金融危机正在向实体经济蔓延，世界经济增速放缓，对我们的影响和冲击正在逐步显现，与我们原有的体制性、机制性、结构性矛盾交织在一起，使发展面临的形势更趋严峻复杂。产业资本观望疑虑加重，招商引资和项目建设难度加大；市场需求低迷，消费能力出现紧缩苗头；企业生产经营受阻，外贸依存度、关联度高的企业更为明显；农民外出务工就业压力加大，给农民收入较快增长带来制约；企业利润空间减小，对石化、纺织和玉米加工等行业影响较大，财政保盘子、保增收难度增大。特别是我们的产业结构不优，支柱产业不强，骨干企业不壮，发展方式粗放，运行质量相对较低，在冲击中可能遇到更多的困难。

同时，更应看到，我们也面临着难得的利好性机遇和挑战性机遇。从利好性机遇上看，国家扩大内需的十条政策措施对我们都十分有利：放松银根、地根，有利于我们破解目前发展的瓶颈问题；加大对农业的支持扶持，有利于我们进一步做强优势；加大城乡基础建设投入，有利于我们加速改变基础建设落后状况；加大对民生的投入，有利于增强我们的财政保障能力；刺激农村消费，有利于我们拓宽农村市场空间。所有这些，对我们都十分难得。从挑战性机遇上看，金融危机也客观上暴露了我们原有体制、机制上的弊端和发展中的薄弱环节，逼着我们要深化改革，调优结构，转变方式，扩大开放。尤其是我们处于内陆地区和产业末端，外向度不高，危机影响的程度相对较小。只要我们冷静应对，辩证思维，抓住机遇，迎难而上，就一定能在冲击中坚强，在挑战中有为。

面对新机遇、新挑战、新要求，保持较快增长最紧要，把千方百计保增长、促发展作为政府工作的首要任务。强化投资拉动最关键，把增加固定资产投资作为保增长的重中之重。转变发展方式最迫切，把优化结构、转变发展方式作为保增长的根本大计。改善民生最根本，把改善民生作为保增长的核心目的。2009年政府工作要以省委经济工作会议和市委二届三次全会精神为指导，深入贯彻落实科学发展观，积极应对世界金融危机带来的风险和挑战，抢抓机遇，突出加快发展，坚持保增长与保民生并重，增速度与提效益统一，上项目与调结构结合，深化改革，扩大开放，转变方式，增强活力，加快推进农区工业化示范区和农业综合开发试验区建设，推动全市经济社会更大发展、更好发展、更快发展。主要预期目标是：地区生产总值增长13%以上；规模以上工业增加值增长12%以上；固定资产投资增长40%以上；社会消费品零售总额增长15%以上；财政一般预算收入增长13%以上；非公经济必保增长15%以上；万元GDP综合能耗下降3％，化学需氧量排放量、二氧化硫排放量分别下降2％和0.5%；城镇居民人均可支配收入和农民人均纯收入分别增长10％和8%；城镇登记失业率控制在4％以内，居民消费价格总水平控制在3%以内，人口自然增长率控制在4‰以内。

具体抓好以下八项重点工作：

（一）狠抓项目开发建设，努力扩大固定资产投资

把上项目作为调结构、增后劲、扩总量、保增长最基础、最必要的工作来推进，不断强化投资对经济增长的拉动作用。一是抢抓机遇，积极争取国家和省的政策投资。抓住国家扩大内需投资导向，重点谋划和争取一批农业、生态、环保、交通、给排水、城市路网、商贸流通以及改善民生和社会公益事业方面的投资项目。对初步拟定的七大类投资101亿元的159个重点项目，进一步精心梳理，抓紧搞好科研论证，争取更多项目列入国家和省投资计划。对去年末已争取到的2.1亿元国家投资，要确保落实落靠，避免因匹配资金不到位等问题而影响项目建设。要大手笔谋划城市建设开发，保持城市基础建设投资增长的强劲势头。突出抓好道路、给排水、垃圾和污水处理等配套工程建设，完善功能，提升品位。年内市本级、肇东、安达三个城市污水处理厂要投入运行，庆安、望奎等七个县（市）的垃圾处理项目要全部开工建设。积极开发清洁能源项目，发展环保建材项目，加强国家级生态示范区和省级自然保护区建设。按照全省建设高等级公路的总体部署，完成好过境高速路的配建任务。二是突出重点，全力以赴招商引资。继续加大招商引资力度。抓住国家实施“九大产业振兴计划”和省“八大经济区”建设的机遇，立足于融入建设、参与建设，推进引资上项目与做大支柱产业、做强特色产业、培育高新产业、壮大财源产业紧密衔接。优化产业布局，延伸产业链条，深度开发品牌产品、高附加值产品，发展循环经济，有的放矢地做强食品、医药、化工、纺织和机电等支柱产业。要强化资源集约、企业聚集、产业集群，抓住金融危机导致一些产业重新“洗牌”，并有意向内陆转移的机遇，加强与发达地区工贸园区协作，努力在成建制、成规模承接产业项目转移上实现突破；进一步加大市本级、肇东、安达三个经济开发区以及青冈玉米、兰西亚麻、明水医药、望奎西郊、庆安绿色食品等产业集中区的建设力度，切实增强项目承载能力。三是完善策略，提高项目建设成效。面对特殊的经济形势，我们的招商引资策略要向国家大的宏观政策上调整，向发挥我市资源和产业优势上调整，向重点区域、重点领域上调整。强化领导招商、全民招商、专业招商，闻风而上，紧盯不放，不断加大工作力度；加强规划招商、战略招商，避免低水平重复建设，避免“半截子”项目、“空转”项目、“摆设”项目，确保建一个成一个。要进一步完善招商引资政策，继续坚持重奖中介人，厚待纳税大户，优化投资环境，集聚人气、商气。全市必保新引进3000万元以上加工项目100个以上，每个县（市）区至少引进亿元以上项目2个，合同引资额和到位资金增长30%以上。

（二）加快发展现代农业，扎实推进新农村建设

保增长的基础在农业。要继续巩固加强农业在保粮食安全、保农民增收、保工业原材料等方面的基础地位，稳步提高农业综合生产能力，推动现代农业建设取得重要进展。全市粮食产能要稳定在200亿斤左右，畜牧业畜禽数量和肉、蛋、奶总量增长10%以上。一是抓好“三个规划”的实施。全面启动240亿斤粮食产能规划、150万吨奶源基地发展规划和1300万头生猪规模养殖规划，加快研究制定农业综合开发实验区实施规划。以“三百工程”为载体，继续抓好水稻、玉米、大豆高产攻关，深入实施庆北绥200万亩水田现代农业示范园区和肇东、青冈等地玉米高产攻关吨粮田园区建设，大幅度提高水稻和玉米单产水平。全市现代农业作业区要发展到30个。加快推进奶牛、生猪规模化饲养的专项重点工程建设，全市新建存栏200头以上标准化奶牛养殖场（区）10个以上，500头以上生猪养殖场（区）100个以上。同时，稳步发展以蔬菜、瓜果、甜菜、亚麻等为主的特色经济作物种植。提升农业产业化发展水平，实现大宗、小宗农产品加工增值，努力建设更具知名度的粮食大市、畜牧业大市、绿色食品大市和农产品精深加工大市。二是大力推进农业标准化生产。用先进科技武装农业，充分发挥公益性科技推广机构、龙头企业、民营科技组织、科技协会和专业大户在农业科技推广中的领军作用，通过各种形式的引带示范，综合搞好各类先进栽培技术、良种应用技术、施肥技术、节水技术、生物技术、纳米技术和农防林截根等技术的推广应用，不断强化科技对农业的支撑作用。全市农业标准化率提高到89%，绿色食品种植面积发展到1500万亩。抓住截根机械纳入国家农机补贴的机遇，推广农防林截根技术80万亩以上。三是加强农业基础建设。千方百计争取国家和省在水利、畜牧、林业、农机、培肥、生态、农业开发、新能源利用、标准化生产等方面的项目和资金支持，增加地方财政投入。重点抓好投资47.6亿元的33个重点农业基础项目的申报和启动实施。加强水利工程建设，继续推进阁山水库项目，争取早日开工建设。基本完成海伦燎原、望奎山头芦、肇东平原等9座中型水库和30个小型水库的消险加固。加强农机化建设，落实好农机补贴和农民购机信贷支持政策，鼓励支持农民、农机大户购买大中型配套农机具，全市新组建投资千万元农机专业合作社3个，农业综合机械化程度达到80%。强化培肥地力，鼓励农民大积大造农家肥，建立专业积肥公司，扩大有机肥源，增加农肥施用量。抓好中低产田改造，全市改造面积要达到200万亩。加强生态建设，完成造林25万亩，治理水土流失面积162万亩。四是扎实推进新农村建设。启动第二批新农村试点工作，争取多方投入，加快发展路、水、电、房、医、教、能、保等农村公共事业。建设通村公路1109公里。新增饮水安全工程300处，解决15万人的饮水安全问题。建设农村沼气池6000处，新增日光节能温室5万平方米。试点村屯“四旁”绿化率达98%。加强农村人才开发，年内培训农民100万人次。五是抓好农民增收。在当前农民工就业压力加大、国内资源性产品价格普遍下行等不利形势下，更加注重抓好农民增收。落实好各项惠民政策，实现政策增收。关注把握农产品价格走势，适时引导农民搞好余粮销售。特别是当前北方多数省份严重干旱，更要注重抓好稳粮增收，鼓励引导农民调优结构，种好效益田。发展特色种养业、加工业，走好一乡一业、一村一品的增收路子。积极扶持返乡农民创业就业，加强就业能力培训，拓展就业渠道。全年内转外输劳动力要稳定在100万人次。

（三）加强指导服务，巩固提高工业经济运行质效

把工业作为保增长的重点，对有下滑趋势的，坚决遏制，并力求有所增长，对有增长潜力的，确保快增长、大增长，实现“以丰补歉”，努力保持工业经济整体上升的势头。一是强化分类指导。对冲击较大的石化、纺织等产业，冷静观察市场变化，强化内部管理，调整产品结构，开拓潜在市场，抓好产销平衡，适时东山再起。对冲击不大的部分食品、粮化工等企业，引导其坚定信心，增加有效供给，拓展发展空间，力求稳中有升。对市场前景看好的医药、机械、建材等行业，引导其多增产、快增产，加强技术创新、管理创新、市场创新，最大限度地扩大市场销售。二是加快在建项目建设进度。对市本级冰雪浆果、北林秸秆发电、肇东延迟焦化、安达亚泰水泥、海伦乳（豆）制品加工、庆安中铁造纸、青冈20万吨淀粉糖、望奎成达肉鸡、绥棱嵩天蛋白质提取、明水格林谷氨酸、兰西医药活性原料加工等60个在建工业项目，要逐个研究加快推进措施，力促快投产、快达产，加快形成新的产能。三是加大扶持力度。对30家重点骨干企业加强调控，积极争取省贴息贷款、技改、创新等专项资金支持，鼓励支持中粮、龙凤、三精、迪龙、佳地、双汇北大荒等具备条件的企业建立研发中心，提升技术水平，增强抵御市场风险的能力。着眼于共渡难关，落实好国家和省对企业出台的扶持政策，对有关税费、基金等能免则免、能减则减、能缓则缓，降低企业经营成本。积极发展各类贷款担保机构，加强银企对接，支持中小企业增加贷款，缓解资金瓶颈制约。帮助重点企业解决用煤、用油和运输等问题，为企业发展提供有利条件。

（四）突出发展现代服务业，推动第三产业优化升级

以发展现代服务业为重点，充分挖掘增长潜力，努力培育壮大新的经济增长点。全市第三产业增加值占GDP比重要提高5个百分点以上。一是大力发展现代物流产业。以市本级、肇东、安达、青冈、望奎、海伦等城市为主要节点，大力推进一些各具特色的专业市场和物流工贸园区建设，着力构建物流支柱产业，打造以中心城市为节点、连接城乡、货畅其流的物流配送运输网络体系。以石化、粮、油、木和绿色食品为重点，大力发展跨区物流、跨国物流。重点建设安达石化、海伦大豆等专业物流中心，建设绥化、海伦、安达等跨省物流节点，支持绥化经济开发区、望奎双汇、肇东大庄园等肉类加工企业建设冷链物流项目，大力扶持安瑞佳、博达等第三方物流企业。全市物流经济对财政贡献率要增长10%以上。二是积极推进旅游业发展。以生态游、田园游、休闲度假游为重点，突出打造寒地黑土特色旅游品牌，进一步开发特色旅游产品。以“全省西部4+2旅游合作”为契机，做强生态休闲、农业观光、民俗文化等三条精品旅游线。全市旅游业收入增长4~5个百分点。三是切实增强商贸产业活力。用足用好国家支持对外贸易发展的政策，加大对青冈龙凤、望奎三合、立达尔、兰西天纤纺、北林健宇、绥棱德志等重点出口企业扶持力度，大力拓展对俄经贸合作，增加有自营出口权企业数量，力争全市进出口总额增长15%以上。加强流通体系建设，发展壮大各类专业市场，在改造提高传统商贸业的同时，加快发展现代商贸流通方式，大力推进“万村千乡市场”和“家电下乡”等工程，继续抓好农村新网工程建设，进一步激活农村市场。四是大力发展社区服务业。利用全民创业的优惠政策，鼓励更多的创业者投身社区服务业，催生拓展服务项目，开办面向社区和乡村的养老托幼、家庭医疗、保洁保绿、区域安全、保养维护等便民服务公司和便民店，增加收入，带动就业。五是有序推进房地产业发展。完善政策调控，制定出台促进房地产开发销售的优惠政策，进一步活跃二级市场和租赁市场，带动一级市场发展，提高普通商品住房、经济适用住房供给比例，促进全市房地产开发平稳健康发展，拉动相关产业发展。

（五）深化改革，增强经济社会发展动力

着眼于增强发展的推动力，认真抓好重点领域的改革。一是大力深化企业改革。紧紧抓住国内外企业战略重组的契机，重点推进企业集团化发展。鼓励域内外关联企业、上下游企业联合重组、联大靠强，实现一体化经营，提高抗风险能力。从区域经济一体化发展的战略布局出发，依托现有的骨干龙头企业，着手组建玉米、乳品、生猪、大豆、医药、硅基材料和亚麻编织加工等产业集团，组建行业协会，加强企业联合合作。要利用产权招商，激活部分倒闭及危困企业存量资产。二是大力深化农村改革。强化公益性农业技术推广机构，对现有的农村“三站”进行改革，年内基本完成乡镇或区域性技术推广中心的组建工作。以规模经营为前提，从政策上积极引导推进农村土地流转，发展多种形式的适度规模经营。发展壮大农村各类新型合作组织，重点扶持一批股份制合作社做大做强，全市规范化农民合作组织要增长10%以上。以项目为载体，整合各类支农资金，集中加快一些重点工程建设。发挥国有粮食企业的主渠道作用，整合域内粮食收购网点，集中打造粮食物流集团。依托大型生资集团，发展生资连锁经营和直销到户，保障农资供应安全。深化乡村两级改革，继续鼓励支持有条件的乡村撤并，支持乡干部到村挂职，以及创办各种服务实体。加强农村“三资”管理，抓好“三清”，增加集体收入。加快推进农牧三场改革，继续推进完善林权改革。三是大力深化金融服务改革。抓住国家金融政策调整的机遇，全力搞活地方金融，充分发挥金融对地方经济发展的支撑作用。加强诚信建设，优化金融环境，严厉打击逃废债行为，完善信用共享平台，对金融失信者实行一票否决。深化金融自身改革，放宽信贷门槛，拓展服务领域，创新金融产品，切实加大对地方经济发展的支持力度。积极创造条件，发展各类融资担保机构，建立银保互动机制。加快发展地方商业银行，大力发展村镇银行、小额贷款公司、农村资金互助社等新型农村金融组织。年内村镇银行或小额贷款公司至少发展2个，农村资金互助社至少发展10个。四是大力深化其他领域改革。认真做好政府机构改革，适时启动事业单位改革，推进运管“三费”、医药卫生体制改革，进一步理顺完善城市监管、城市供排水、城市社区和社会保障管理等方面的体制、机制问题。

（六）提升综合实力，进一步加快市本级发展

今年，市本级发展，突出扩大经济总量，做强主导产业；突出完善城市功能，重点实施路网覆盖、精品绿化、棚改安居、管理升级“四大工程”；突出改善民生，加强社会建设，提高中心城市建设水平。全口径财政收入和一般预算收入分别增长13%和14%；规模以上工业增加值、利税分别增长12%和13%。具体要抓好十项重点工作：一是重点抓好开发区建设。深化招商引资，着力做大做强食品医药、硅基材料、机械电子、现代物流等支柱产业。巩固发展现有企业，加快在建项目进度，力推已入区的硅加工项目如期完成一期建设，继续大力引进硅产品深加工项目，力争5年内把硅产业打造成产值超百亿、利税超10亿的市本级立市产业。通过加大招商引资力度，发动全民招商，力争今年新上超亿元项目3个以上，并有一批中小项目在开发区落户。加强开发区基础建设，增加投入。抓住新一轮土地利用规划修编的机遇，增加用地指标，提高项目承载能力。二是抓好道路建设。投资3000万左右，打通、拓宽、新建主次干道、巷道20条，抓好人和西街、福和西街等4条道路4万平方米人行道铺装，改造绥望路、紫来街、双桥街、西环等14条排水网线，完成绥望、绥兰等重点出城口的改造。三是抓好绿化美化。投资5000万元，开展道路、校园、小区、庭院绿化，实现主次干路基本绿化，城市建成区绿化覆盖率达30%以上。四是抓好精品工程建设。改造提升一批具有观赏性、实用性，体现生态性的标志性工程，把中兴路打造成“精品商业街”，把康庄路建设成生态长廊，把碧桂园、福乾花园、颐和苑建成高档、亮点小区。五是抓好安居工程建设。拆迁改造棚户区20万平方米以上，完成建筑面积40万平方米以上，新建廉租住房12万平方米以上，房地产总计开发面积80万平方米以上。六是抓好休闲便民设施建设。改造水利排灌、教堂南广场等广场公园4处，改造旱厕15个，新建水冲公厕5个。七是抓好集中供热建设。积极筹建城区第二热源，争取管道燃气项目立项、施工，新增集中供热能力50万平方米,力争完成70万平方米。八是抓好社区建设。新建3个400平方米、5个300平方米社区服务站，2个1000平方米街道社区服务中心。九是抓好重点公益服务项目建设。争取招商建设星级宾馆和经营面积3万平方米左右的大型商场。积极创造条件，加快建设数字化城管系统，启动中国绿色产品特供中心绥化分中心建设。地下商业街 “十一”前投入使用。十是抓好城市管理。以创建优美环境、优良秩序、优质服务“三优”文明城创建为载体，理顺机制，完善体制，落实责任，从根本上解决城市管理脏乱差问题。

（七）加强社会建设，着力保障和改善民生

积极对上争取支持，千方百计增加投入，继续加大民生事业力度。一是大力推进就业再就业。深入实施创业带动就业战略，加强就业援助，增加小额贷款支持，大力开发公益性岗位，年内新增就业再就业5万人以上。鼓励支持遇到困难的企业妥善安置待岗工人。二是全面推进保障性住房建设。完成棚户区改造拆迁面积119万平方米，廉租住房保障29870户，其中实物配租7122户，租赁补贴22748户。改造农村泥草房5.25万户，倒危房2500户。探索“农村公屋”、“五保小区”、“零租房”等农村住房保障实现形式。三是强化社会保障。启动新型农村养老保险试点，力争试点参保率达到15%以上。全面启动城镇居民医疗保险，推进“五险”统筹扩面。加强社会救助，争取新建4个县级中心敬老院、1处儿童福利院和流浪未成年人救助保护中心。扩大“双低”人群医疗救助保险覆盖面，城市低保对象参保面要达到90%，城市低收入人群参保面要达到50%以上。深入落实好妇女儿童发展规划。四是扎实抓好扶贫解困。深入推进农村万名妇女解困、千会带万户行动，切实抓好扶贫开发整村推进和城市职工解困工作，确保城乡7万人脱贫。五是改善医疗卫生服务质量。加强城乡卫生服务网络建设，完成30个乡镇卫生院改造，社区医疗机构及服务覆盖率提高到85%。做好优生优育工作，提高计生服务水平。六是加强食品药品和重要农资质量监管。严厉打击制假贩假行为，坚决遏制有毒有害食品、药品流入市场。严防假种子、假农药、假化肥等坑农害农问题发生。七是推动教育事业科学发展。统筹城乡教育发展，整合优化城乡教育资源。增加投入，落实好标准化学校建设规划，改造建设标准化学校30所，新建职教实训基地6处。大力实施素质教育，努力提高教育教学质量。落实各项教育惠民政策，保证进城农民工子女就近就学。八是积极推进文体惠民工程。升级改造1个市级、5个县级文化信息资源共享中心，乡镇综合文化站覆盖率提高到48%，新建100处基层文化服务点和农家书屋；完成全民健身工程80处以上；推进广播电视数字化网络化进程，新发展数字电视2万户。加强社区活动场所建设，建成1000平方米以上的县级社区服务中心7个，500平方米以上的街道社区服务中心10个，300平方米以上的社区服务站40个，精品社区20个，社区办公用房自有率达到80%。九是推进城镇集中供水、供热工程建设。加强城镇供水管网改造和二次提水设施改造，推进供水计量改造，整合供水资源，扩大全天候供水面积。推进集中供热项目建设，新增集中供热能力150万平方米，集中供热率提高到53%。十是全力抓好“平安绥化”建设。加强信访工作，及时解决好群众反映强烈的突出问题，化解矛盾，消除不稳定因素。加强社会治安综合治理，严厉打击各种违法犯罪行为，确保一方平安。加强安全生产监管，坚决遏制重特大事故。加强公共安全管理，建立健全应急突发事件管理和信息反馈机制。加强市场价格监管，发挥好价调基金的调控作用。民生就是民心，民心就是使命。我们一定在保障民生基础上，更加切实改善民生，不论今年财政增收多少，对民生投入只能增加，不能减少，拓展民生领域，提高保障水平，切实让更多的惠民成果书写在绥化大地上，铭刻在老百姓的心坎上。

（八）加强政府自身建设，全力推进工作落实

新的形势，新的任务,对政府工作提出了新的、更高的要求。我们要加大思想解放的力度。坚持在保增长上解放思想，在重民生上解放思想，在破解难题上解放思想，不论困难多大，压力多大，都要坚持更大更快更好发展的决心不动摇，善于突破，勇于创新，敢于负责，切实把保增长落实到增速提效上，把保民生体现到老百姓的笑脸上，把解难题转化为坚定各级干部的信心上。加大科学管理的力度。加强对各级公务员的教育培训和管理，提高服务于科学发展的能力和本领。完善绩效考核，对县（市）区要既考核相对增长量，又要考核绝对增长量，对市直部门突出大事实事的考核，突出争取项目资金多少的考核。充分发挥审计的作用、监察的作用、督查的作用和检查的作用，对执行不力、假公济私、推诿扯皮、玩忽职守等行为严肃追究责任，实施行政问责。加大作风转变的力度。坚持鼓实劲不务虚功，求实效不图虚名，踏实地不浮不躁，任苦干不事张扬。深入基层、深入企业、深入项目建设现场，一户一户地帮助想对策、渡难关，一个环节、一个环节地抓推进、抓落实。对定下来的事情，就要抓出头，抓到底，决不能让计划变成空话，规划变成图画。加大环境优化的力度。深入开展“发展环境优化年”活动。规范行政收费、行政审批，从解决以费谋利、趁乱收费问题抓起，认真清理和削减行政收费项目，转变职能，提高效率。深入推行首问负责制、公开办事制、限时办结制、服务承诺制，进一步强化马上办办公室的作用，对顶着不办、拖着缓办的“一票否决”。加大廉政勤政的力度。坚持科学执政，依法行政，民主施政，紧紧依靠市委领导，主动接受人大的法律监督、政协的民主监督、媒体的舆论监督和广大人民群众的监督。加大推进办事公开化力度，建设阳光政府，把工程招投标、上项目、房地产开发、土地出让、市政工程、人事管理、资金管理全部公开化，在阳光下公开透明操作。进一步健全各项规章制度，完善决策程序，筑牢反腐倡廉防线。大力开展节约节能活动，树牢过紧日子的思想，厉行节约，降低成本。我们还要进一步加强政府班子自身建设。我将和各位副市长及其他政府组成人员，时刻牢记使命、不负人民重托，继续同心协力，自我加压，励精图治，奋发图强，切实做到对人民负责信得过，受人民监督叫得硬，让人民满意靠得住！

各位代表！让我们在省委、省政府和市委的坚强领导下，深入学习实践科学发展观，抢抓机遇，奋力进取，同心同德，扎实苦干，为完成今年的各项目标任务，实现全市经济社会更大发展、更好发展、更快发展而努力奋斗！
